# From Alzheimer’s Disease to Anxiety, Epilepsy to Schizophrenia: A Comprehensive Dive Into Neuro-Psychiatric Disorders

**DOI:** 10.7759/cureus.58776

**Published:** 2024-04-22

**Authors:** Han Grezenko, Zarin Nudar Rodoshi, Ciara S Mimms, Muhammad Ahmed, Astrit Sabani, May Su Hlaing, Biniyam J Batu, Muhidin I Hundesa, Biruk D Ayalew, Abdullah Shehryar, Abdur Rehman, Ahmad Hassan

**Affiliations:** 1 Medicine and Surgery, Guangxi Medical University, Nanning, CHN; 2 Translational Neuroscience, Barrow Neurological Institute, Phoenix, USA; 3 Medical Education, Mymensingh Medical College, Dhaka, BGD; 4 Medicine, St. George's University, St. George's, USA; 5 Psychiatry and Behavioral Sciences, Dow University of Health Sciences, Karachi, PAK; 6 Geriatrics, United Lincolnshire Hospitals NHS Trust, Boston, GBR; 7 General Practice, St. Paul's Hospital Millennium Medical College, Addis Ababa, ETH; 8 Medical Services, Federal Democratic Republic of Ethiopia Ministry of Health, Addis Ababa, ETH; 9 Internal Medicine, St. Paul's Hospital Millennium Medical College, Addis Ababa, ETH; 10 Internal Medicine, Allama Iqbal Medical College, Lahore, PAK; 11 Surgery, Mayo Hospital, Lahore, PAK; 12 Internal Medicine, Mayo Hospital, Lahore, PAK

**Keywords:** schizophrenia, epilepsy, clinical anxiety, autism spectrum disorder and anxiety disorder, depression

## Abstract

This comprehensive narrative review endeavors to dissect the intricate web of neuropsychiatric disorders that significantly impact cognition, emotion regulation, behavior, and mental health. With a keen focus on Alzheimer's disease (AD), anxiety disorders, epilepsy, schizophrenia, and autism spectrum disorder (ASD), this article delves into their underlying mechanisms, clinical presentations, diagnostic challenges, and therapeutic interventions. Highlighting the considerable disability and societal costs that these conditions impose, it reflects on the over six million individuals grappling with Alzheimer's, the 19 million American adults living with anxiety disorders, the three million with epilepsy, and the global reach of schizophrenia affecting approximately 20 million people. Furthermore, it examines the emerging landscape of ASD, noting the escalating diagnosis rates and the pressing need for innovative treatments and equitable healthcare access. Through a detailed exploration of current research, technological innovations, and the promise of personalized medicine, this review aims to illuminate the complexities of these conditions, advocate for early intervention strategies, and call for a unified approach to tackling the multifaceted challenges they present. The ultimate goal is to inform and inspire healthcare professionals, researchers, and policymakers to foster advancements that improve outcomes and quality of life for individuals affected by these profound neuropsychiatric disorders, steering towards a future where these conditions are no longer insurmountable.

## Introduction and background

Neuropsychiatric disorders make up a broad class of neurological and psychiatric illnesses impairing cognition, emotion regulation, behavior, and mental health. Prominent examples include dementia syndromes, anxiety disorders, epilepsy seizures, and psychotic conditions such as schizophrenia. These diseases confer substantial disability on patients and families alongside soaring societal costs. In 2022, within the United States alone, over six million individuals suffered from Alzheimer’s disease (AD), while healthcare expenditures reached $321 billion [1]. Annual estimates further quantify over 19 million American adults living with anxiety disorders [2] and three million with epilepsy [3]. Globally, schizophrenia affects approximately 20 million people [4]. This review synthesizes key facets across four major neuropsychiatric underlying mechanisms, clinical presentations, and diagnostic and therapeutic considerations while deeply analyzing interrelationships. It also comprehensively explores frontiers such as personalized medicine, technology innovations, and ethical dimensions that promise to transform future management approaches for these profound conditions.

The primary objective of this article is to elucidate the complex etiology, pathophysiology, diagnostic challenges, and current therapeutic strategies associated with a range of neuropsychiatric disorders, including AD, anxiety disorders, epilepsy, schizophrenia, and autism spectrum disorder (ASD). By synthesizing the latest research findings and clinical insights, this review offers a multidimensional understanding of these conditions, highlights their significant impact on individuals and society, and explores the potential of emerging treatments and personalized medicine. Furthermore, it aims to bridge knowledge gaps, advocate for integrating innovative healthcare solutions, and underscore the critical importance of early intervention and access to care. This narrative endeavors to inform and influence healthcare professionals, researchers, and policymakers, contributing to improved healthcare outcomes and fostering a collaborative approach to addressing the complexities of neuropsychiatric disorders.

## Review

AD

AD represents the most common cause of progressive neurocognitive decline, leading to dementia, particularly affecting older adults. The course proves irreversible once initiated. Hallmarks of AD neuropathology include extracellular amyloid-beta plaque deposits and intracellular neurofibrillary tangles containing aggregated, hyperphosphorylated tau protein [5]. Familial, early-onset AD results from gene mutations coding amyloid precursor protein and presenilins involved in amyloid processing. However, in late life, 95% of cases occur sporadically without clear inherited genes. These likely arise through intricate interactions between candidate risk genes regulating inflammation, cholesterol metabolism, insulin signaling, and vascular factors alongside environmental exposures such as diet, exercise, education, and toxic elements [6].

Neurodegeneration and Clinical Presentation in AD

As AD pathology accumulates, neurons progressively dysfunction and die, first in medial temporal lobe memory structures such as the hippocampus and then spreading to diffuse association cortices. Consequent deficits manifest insidiously but relentlessly in memory, thinking, behavior, and functional competence. Although some individuals exhibit pure cognitive impairment, others also develop psychiatric symptoms such as depression or psychosis. Diagnostic criteria center on clinical history corroborated by caregiver reports and objective cognitive assessments. Structural neuroimaging techniques visualizing medial temporal lobe atrophy and diffuse cortical thinning increase certainty. No definitive cure currently exists, but acetylcholinesterase inhibitors and memantine may provide temporary cognitive/functional stabilization by enhancing neurotransmitter signaling among surviving neurons [7].

Advancements in AD Research: Targeted Therapies and Multimodal Approaches

**Figure 1:**
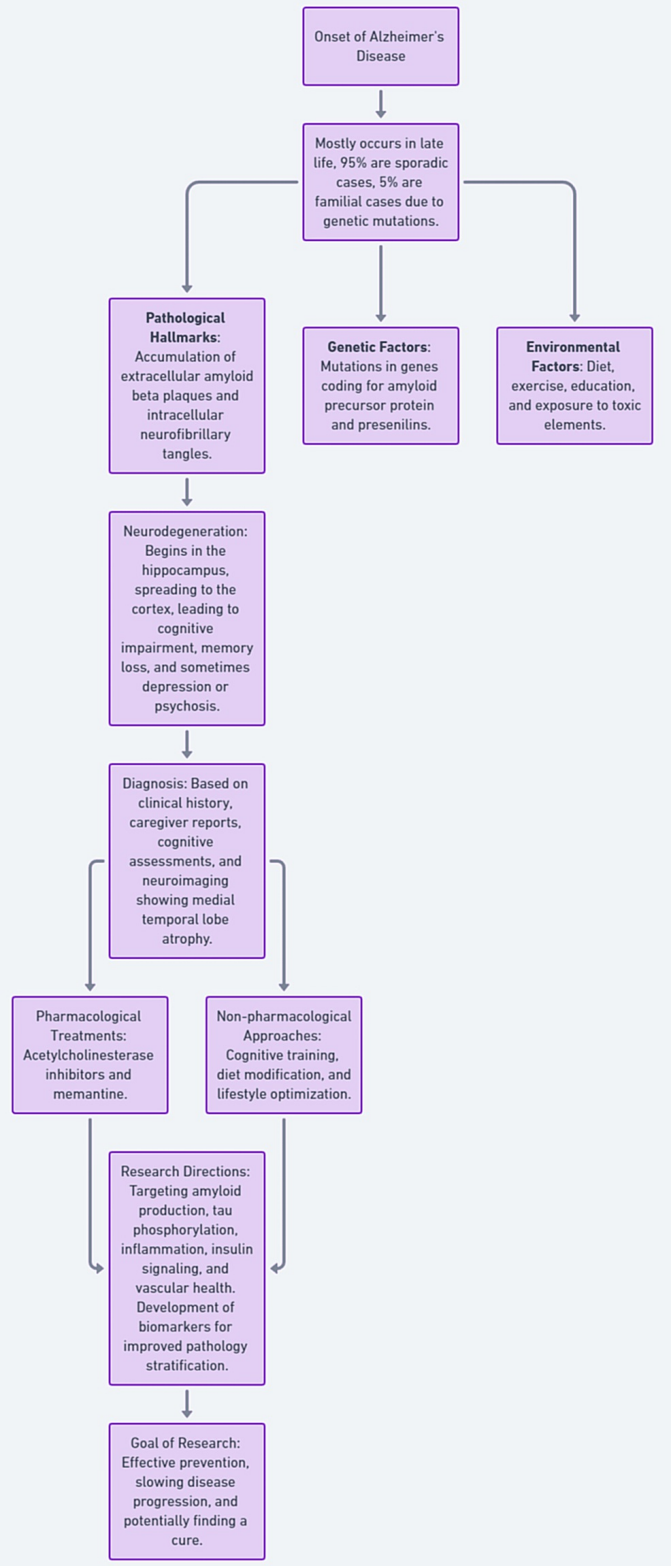
A comprehensive flowchart of Alzheimer's disease progression and management strategies. The image was created by the authors.

Myriad clinical trials targeting putative AD pathophysiology are underway seeking to slow disease progression. Agents decreasing amyloid production or aggregation, preventing tau phosphorylation, dampening inflammation, and optimizing insulin signaling and vascular health demonstrate theoretical promise [8]. Combination treatment strategies also arise, guided by improved biomarkers stratifying underlying pathology. Beyond drugs, non-pharmacological approaches such as cognitive training, diet modification, and biomarker-guided lifestyle optimization also expand the options for preserving neurological function [7]. Despite the profoundly expanding aging population conferring greater disease prevalence, such steady research progress, fuels hopes that effective prevention and treatment lie on the horizon. A comprehensive flowchart is provided in Figure 1.

Anxiety disorders

Anxiety disorders, encompassing generalized anxiety, social phobia, and panic attacks, are characterized by overwhelming fear and worry. These conditions, difficult to control and often disproportionate to actual threats, disrupt daily life and affect over 30% of individuals, with around 19 million American adults suffering annually [2]. Stress, arising from various sources, combines genetic predispositions and neurobiological mechanisms to exacerbate these disorders.

Insights Into Neurobiology and Symptomatology

Prominent anxiety pathways center on the hypothalamic-pituitary-adrenal axis governing stress hormone response, prefrontal cortex executive functioning, and fear circuitry focused on the amygdala [9]. Neural network disturbances manifest in abnormal information processing biases skewed towards increased threat detection and heightened emotional reactivity. Core symptoms span psychological and somatic spheres, including persistent worrying, panic attacks, avoidance behaviors, muscle tension, insomnia, gastrointestinal upset, and more. The presentation further depends on specific diagnoses ranging from generalized anxiety to phobias to post-traumatic stress. Thorough clinical history and targeted symptom rating scales facilitate accurate diagnosis.

Effective Management Strategies for Anxiety Disorders

**Figure 2:**
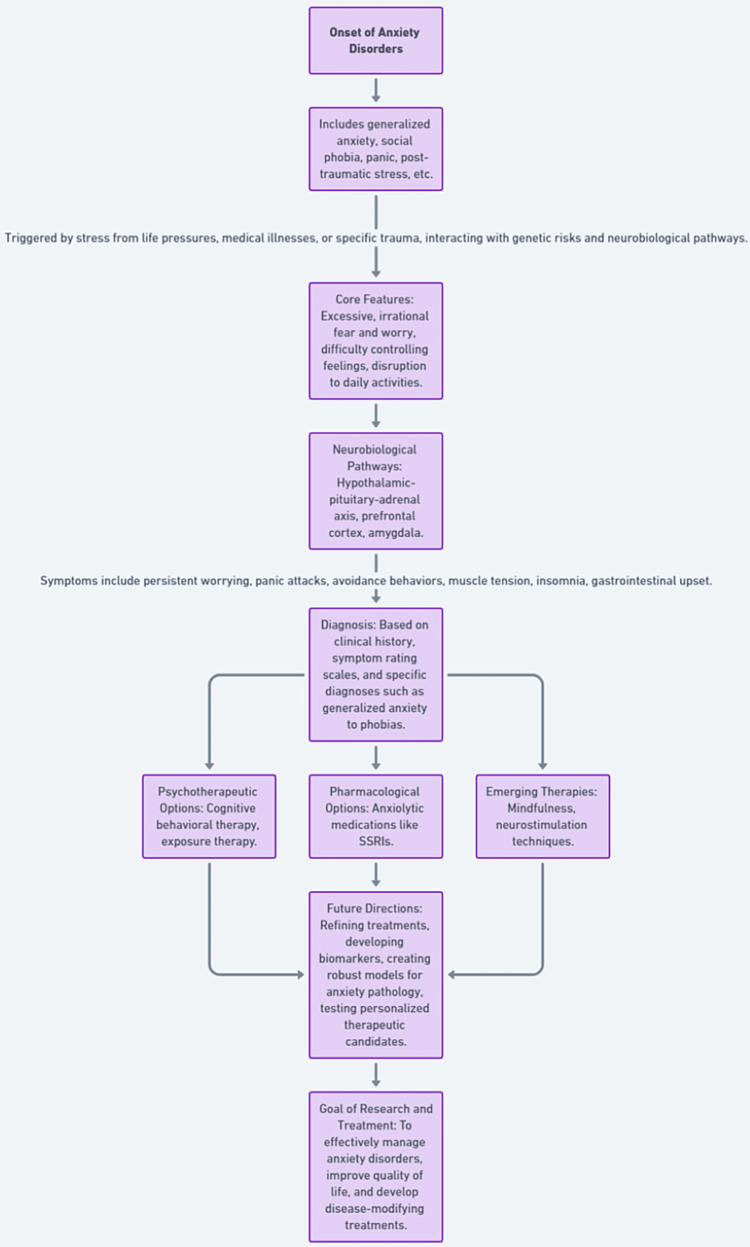
A detailed flowchart of the progression, diagnosis, and management of anxiety disorders. The image was created by the authors.

Once identified, a range of psychotherapeutic and pharmacological options effectively manage anxiety for many patients. Cognitive behavioral therapy builds mental skills, decreasing excessive fears. Exposure therapy extinguishes trigger response through gradual acclimation. Anxiolytic medications such as selective serotonin reuptake inhibitors alleviate symptoms long term. Research continues refining mindfulness approaches leveraging meditation to reduce reactivity. Neurostimulation techniques also show promise. Understanding neurobiological mechanisms enables stratifying interventions based on disturbance profiles, while biomarker development and imaging quantitatively track treatment efficacy at the brain circuit level [10]. Still needed are more robust models fully capturing complex anxiety pathology and testing new personalized therapeutic candidates in humans to transform disease-modifying options. A comprehensive flowchart is provided in Figure 2.

Epilepsy

Epilepsy, a diverse spectrum of disorders, is characterized by frequent, spontaneous seizures due to abnormal neural activity. This condition is classified into various types, distinguished by their causes - genetics, brain injuries, and seizure locations. Its heterogeneity complicates diagnosis, necessitating detailed clinical evaluations, EEGs, and patient histories to rule out other conditions, such as syncope or psychogenic non-epileptic spells [11]. Diagnostic processes are supported by genetic testing and neuroimaging, which illuminate the specific pathology and anatomy involved, thereby informing the management and prognosis of epilepsy. This comprehensive approach underscores the complexity of epilepsy, emphasizing the need for precise identification and tailored treatment strategies.

Current Landscape and Challenges in the Treatment of Epilepsy

Available treatment options have not substantially advanced in decades, centering on anticonvulsant medications controlling seizures paired with lifestyle adjustments and treatment of any underlying disorders. Dozens of agents reduce excitation or enhance inhibition across neurotransmitter systems, employing complex pharmacodynamic interactions and pharmacokinetic optimization to balance efficacy with side effects. Intractable cases require alternative interventions such as a ketogenic diet, implantable devices delivering responsive stimulation, or respective epilepsy surgery removing seizure foci [12]. Despite therapeutic options, significant disability, and reduced quality of life, the persist-emphasizing need to understand mechanisms further and expand modalities customized to individual patients.

Innovative Strategies in Epilepsy Treatment

**Figure 3:**
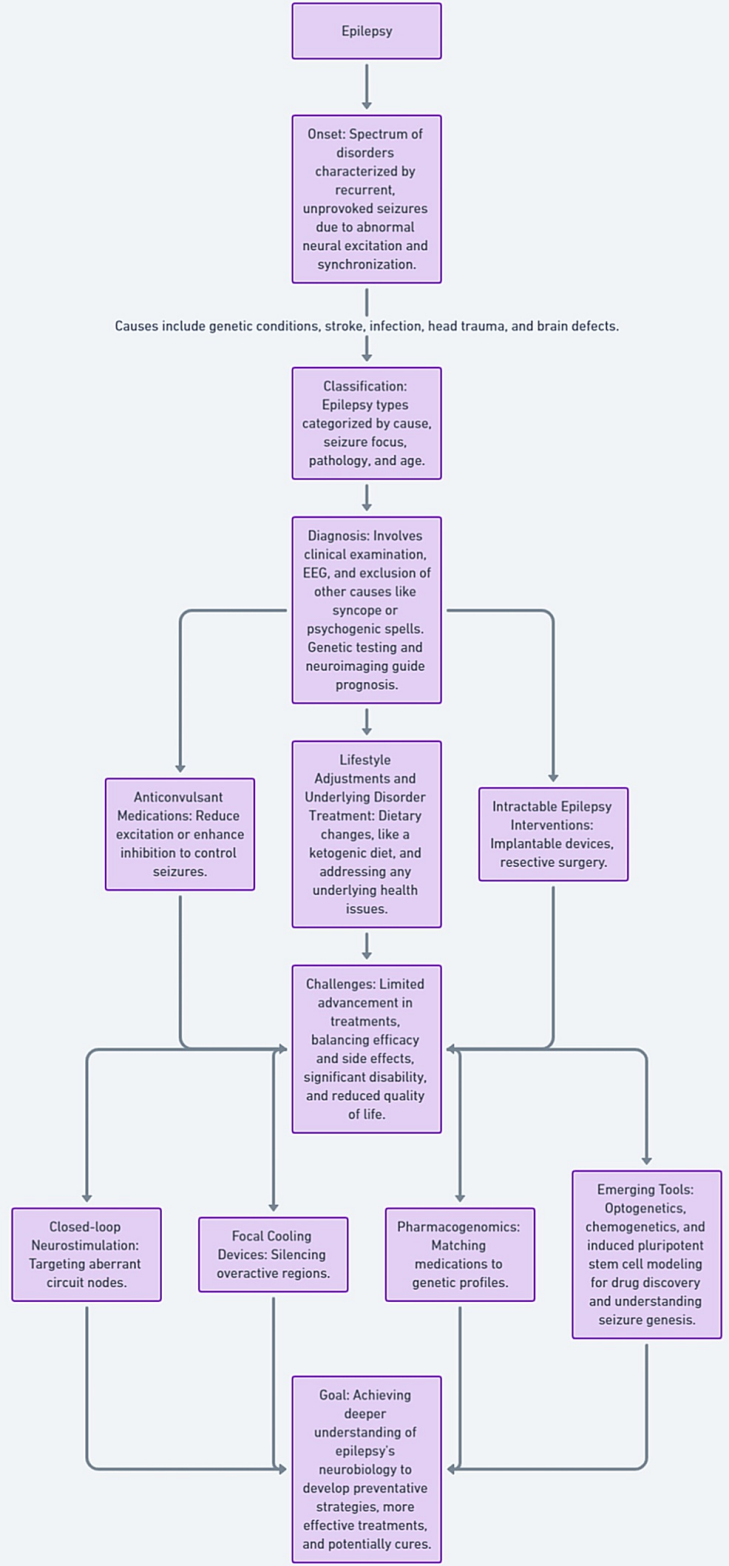
A comprehensive flowchart from onset to innovative treatments and goals. The image was created by the authors.

Several promising approaches gaining traction include closed-loop neurostimulation systems mapping and then targeting aberrant circuit nodes; focal cooling devices reversibly silencing overactive regions; and pharmacogenomics matching medications to patients’ genetics predicting drug response and tolerability [13]. Still critically needed is cracking the neurobiology of how healthy neural tissue transitions to generating recurrent, spontaneous seizures. With enhanced clarity of pathogenesis, emerging tools such as optogenetics and chemogenetics could someday precisely correct network dysfunction while induced pluripotent stem cell modeling offers revolutionary platforms for efficient drug discovery-perhaps even uncovering preventative strategies or radical cure options moving forward. A comprehensive flowchart is provided in Figure 3.

Schizophrenia

Schizophrenia represents one of the most profoundly disabling severe mental illnesses, traditionally categorized as a psychotic disorder given hallmark symptoms violating reality through distorted thoughts and perceptions. Positive symptoms classically involve hallucinations, often auditory voices, but potentially spanning sensory modalities and delusions reflecting fixed false beliefs impervious to reason. Negative attributes include diminished motivation, speech output, emotional expression, social withdrawal, and apathy. Cognitive dysfunction also proves ubiquitous, impairing attention, memory, and executive functions. Schizophrenia usually manifests between ages 16-30 years with ~1% lifetime incidence [4]. Men tend towards earlier onset and greater neurobiological deficits overall.

Unraveling the Complexities of Schizophrenia Pathology

Despite decades of investigating underlying schizophrenia pathology, origins remain opaque given the complexity of variable symptomatology, clinical course, and treatment response. Still, dopamine overactivity, particularly in mesolimbic networks, regulates salience signaling and predominates in models, although glutamate dysfunction also contributes [14]. Instead of defined lesions, microcircuit abnormalities involving excitatory/inhibitory tone modulation are associated with positive and negative symptoms. No definitive confirmation by histology or imaging exists, consistent with core features arising from distributed dysregulation patterns traversing cortical and subcortical structures [15]. Environmental impacts further obscure etiological origins.

Advancements in Schizophrenia Management

Presently, managing schizophrenia centers around dopamine-blocking antipsychotic medications attenuating acute psychosis and reducing relapse risk, while psychosocial approaches promote functioning [16]. However, agents prove only partially effective for many, with tolerability frequently limited by neurological side effects, highlighting the need for better options. Still, personalized medicine advances now enable grouping patients with distinct symptom and functional impairment profiles and then matching specific interventions or even predicting their responses based on genetics and bioinformatics classifiers [17]. Parallel revolutionary advances employing stem cell and genetic engineering models provide unprecedented ability to construct patient-derived neurons recapitulating schizophrenia-associated mutations [16]. Such cells present flexible platforms investigating disease biology and screening therapeutic candidates, offering hope that precision treatments may soon transition from detached wishful thinking to clinical reality.

ASD

ASD represents a complex neurodevelopmental challenge marked by difficulties in social interaction and communication and a pattern of restricted and repetitive behaviors. Characterized by a wide spectrum of symptoms and abilities, ASD's impact on individuals varies significantly, necessitating early identification as outlined in the DSM-5 [18]. Multifactorial etiology involves a blend of genetic predispositions and environmental factors contributing to its diverse manifestations. Over recent decades, there has been a noticeable surge in ASD diagnoses globally, attributed more to heightened awareness, expanded diagnostic criteria, and improved detection methods rather than an actual increase in occurrence. Currently, about one in 54 children in the United States are identified with ASD [19], highlighting it as a growing public health issue recognized by the World Health Organization. This rising prevalence amplifies the call for enhanced research, resources, and public health initiatives to support the ASD community effectively [19].

Neurobiology and Pathophysiology

The neurobiology and pathophysiology of ASD are marked by intricate changes in brain structure, connectivity, and neurotransmitter dynamics, underpinning the diverse symptoms of the condition [20]. Advances in neuroimaging have uncovered atypical developments across critical brain regions responsible for social cognition and communication, such as the prefrontal cortex, amygdala, cerebellum, and temporal lobes, which likely influence the noted challenges in social interaction and sensory processing in individuals with ASD. An observed dysregulation in brain connectivity, characterized by patterns of both hyperconnectivity and hypoconnectivity, points to a fundamental imbalance in how neural networks integrate and segregate information, contributing to ASD's hallmark sensory sensitivities and communication difficulties [21]. Moreover, discrepancies in neurotransmitter systems, notably within the gamma-aminobutyric acid (GABAergic), serotonergic, and glutamatergic pathways, reveal a complex interplay of factors, disrupting the balance between neural excitation and inhibition [22]. These neurobiological insights emphasize the complexity of ASD, showcasing a disorder shaped by a broad array of neural circuitry and neurotransmitter anomalies that drive its varied clinical manifestations.

Challenges and Future Directions

Diagnosing and managing ASD present significant hurdles, especially regarding timely, accurate diagnosis and fair access to care. Early detection is crucial for effective intervention, yet the variability in symptoms and a lack of specialists often delay diagnosis [23]. Moreover, socioeconomic, geographical, and racial disparities hinder equal access to services, affecting the quality of care and outcomes for ASD individuals. Addressing these issues demands more inclusive health policies. On the horizon, research and therapy for ASD are advancing towards closing these gaps with innovative treatments such as neurofeedback, virtual reality, and pharmacological enhancements, alongside the promising prospects of genomics and personalized medicine to pinpoint risk factors and tailor treatments. Developing and validating ASD biomarkers remains a pivotal challenge, potentially transforming early detection and intervention [24]. Early and targeted support is paramount for improving cognitive and social development outcomes. Ongoing research into ASD's complexities must translate into practical, equitable healthcare solutions, ensuring comprehensive support for all affected individuals to reach their fullest potential.

Comparative analysis and interrelations

Upon analyzing neurobiology and treatment considerations between highlighted psychiatric and neurological conditions, notable interrelationships emerge by which learning from one domain contextualizes others. For instance, anxiety and depression frequently accompany epilepsy and schizophrenia while independently raising dementia risk over the lifespan through synergistically disrupting regional brain plasticity essential for cognitive resilience [25]. Schizophrenia shares genetic associations, including L-type calcium channel signaling genes, with bipolar disorder, which manifests episodic disturbances in mood, activity, and psychosis-hinting at molecular commonalities across these diagnoses [26]. Further, childhood seizures often present years before early-onset AD, indicative of shared pro-excitatory pathological processes aberrantly activating microcircuits whose progressive failure enables accumulating AD pathology [27].

**Figure 4:**
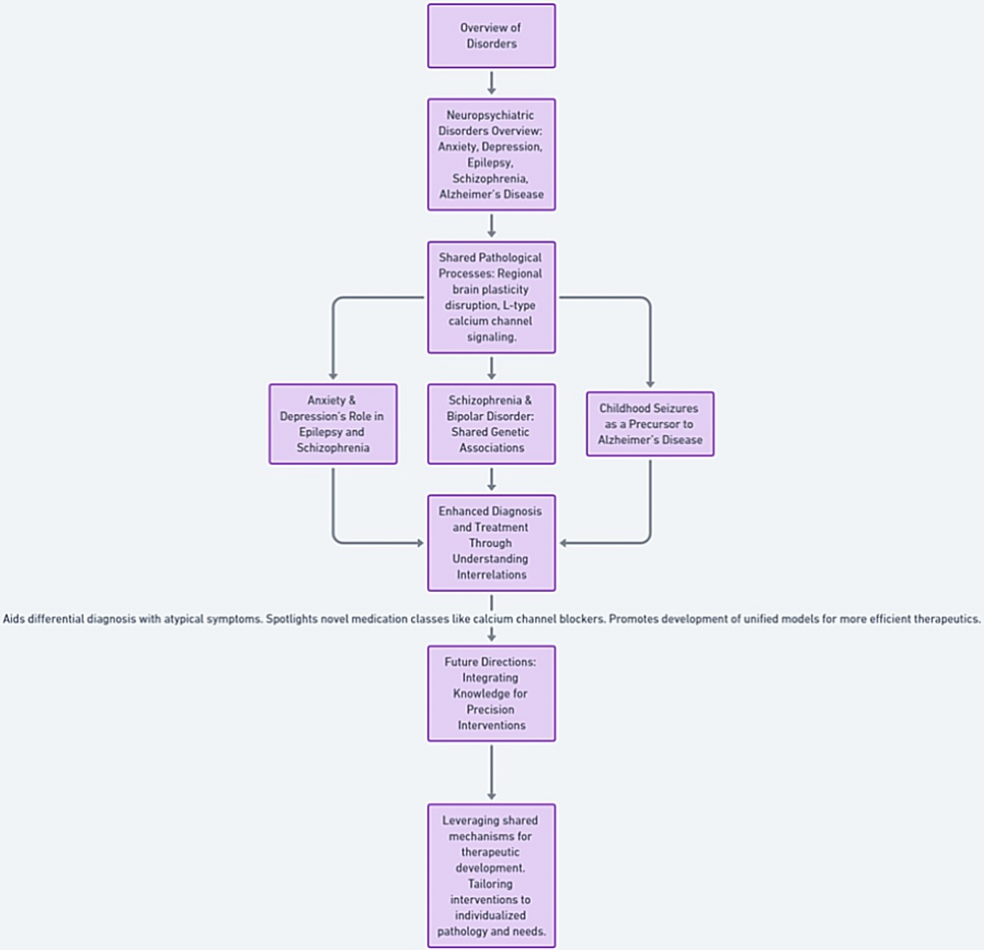
The flowchart representing the interrelations among neuropsychiatric disorders. The image was created by the authors.

Understanding the intersections of neurological and psychiatric disorders for enhanced diagnosis and treatment and appreciating such correlations and distinctions between neurological and psychiatric illnesses aid differential diagnosis when atypical symptoms cloud clinical presentations. It further spotlights potentially fruitful novel medication classes such as calcium channel blockers that could simultaneously alleviate facets of multiple disorders. Even more exciting are revelations that distinct conditions may arise from similar pathological processes manifesting divergently based on factors such as genetics and anatomical vulnerability. Building unified models leveraging shared mechanisms promises increased efficiency in developing therapeutics. Integrating knowledge across classically defined diagnostic categories will prove essential for delivering precision interventions tailored to individualized pathology and personalized needs. A comprehensive flowchart is provided in Figure 4.

Challenges and future directions

Formidable obstacles exist on the path toward preventing and optimally treating neurological and psychiatric conditions. Contention persists around clearly defining normal aging versus pathological processes, resulting in clinical dementia syndromes [28]. Challenges integrating tremendously heterogeneous individual data towards cohesive explanatory models affect most domains -amplified by gaps even defining fundamental hallmarks of diseases such as schizophrenia after decades of investigating pathophysiology. This area should join almost all other medicine in incorporating more patient advocacy and representation in research efforts. Most central nervous system drug developments, both small molecule and biologic, demonstrate extensive failure rates when models only partially recapitulate complex human illnesses and then optimistically attempt leaping straight into patient trials [29]. Preclinical sciences need further development to improve preparedness to support clinical investigations.

Beyond scientific frontiers, delivering innovative interventions once discovered requires confronting ethical debates surrounding patient capacities for consent across situations such as acute psychosis or progressive dementia syndromes and managing risks for coercion, exerting undue influence on participation choices during perceived power differentials in psychiatric care relationships while working to eliminate pernicious social stigmas disenfranchising those battling psychological or neurological conditions [30]. Beyond transforming direct care, supporting patients and families experiencing neurological or psychiatric challenges demands evolving public policies around healthcare access and affordability, workplace accommodations, community integration assistance, and caregiver wellness.

Despite sobering challenges, room for optimism persists, grounded in visions where revolutionary scientific technologies could suddenly crack mysteries barely yielding ground in decades. Genetics and bioinformatics convergence enables personalized stratification and targeting of pathology, as already realized in fields such as oncology, but it awaits full actualization for central nervous system applications. Stem cell engineering uniquely offers patient-derived human neuronal models manifesting disease phenotypes in vitro and then efficiently screening drug candidates or even elucidating underlying genes and biological pathways for intervention. These could selectively correct core physiological disturbances. Gene therapies utilizing cutting-edge editing techniques, vector delivery systems, and neuronal interface devices represent promising options.

Ultimately, integrated basic, translational, and clinical research across academic, government, and industry groups offers the greatest prospects for conquering diseases currently incurable. However, essential too is empowering patients themselves steering collaborative efforts. Such comprehensive, multifaceted initiatives focusing expertise and resources in unified missions promise hope that neurological and psychiatric disorders need not remain permanently untreatable mysteries barely managed, but one day soon could transition towards optimized outcomes benefitting all whose lives they touch.

## Conclusions

In this comprehensive exploration of neuropsychiatric disorders, from the intricacies of AD to the complexities of schizophrenia and beyond, we have traversed the broad spectrum of challenges and advancements that define the current state of neurological and psychiatric healthcare. This review has illuminated these conditions' profound impact on individuals and society and highlighted the critical importance of early diagnosis, innovative treatments, and the need for accessible care. As we look to the future, it is evident that the path toward transformative progress in managing these conditions lies in fostering integrated research and clinical collaborations, embracing patient-centered approaches, and leveraging technology and personalized medicine. By uniting efforts across disciplines and stakeholders, we stand on the precipice of a new era in mental healthcare marked by hope, innovation, and a deeper understanding that promises to alleviate the burden of neuropsychiatric disorders for individuals around the globe. This vision, both ambitious and essential, beckons us to a future where the mysteries of the mind are managed and mastered, offering brighter prospects for all affected.

## References

[1] The Alzheimer's Association (18). 2022 Alzheimer's disease facts and figures. Alzheimers Dement.

[2] (2024). Anxiety disorders. https://www.nimh.nih.gov/health/topics/anxiety-disorders.

[3] (2024). Epilepsy Data and Statistics. https://www.cdc.gov/epilepsy/data/index.html.

[4] Simeone JC, Ward AJ, Rotella P, Collins J, Windisch R (2015). An evaluation of variation in published estimates of schizophrenia prevalence from 1990─2013: a systematic literature review. BMC Psychiatry.

[5] Yu L, Boyle P, Schneider JA, Segawa E, Wilson RS, Leurgans S, Bennett DA (2013). APOE ε4, Alzheimer's disease pathology, cerebrovascular disease, and cognitive change over the years prior to death. Psychol Aging.

[6] Livingston G, Huntley J, Sommerlad A (2020). Dementia prevention, intervention, and care: 2020 report of the Lancet Commission. Lancet.

[7] Cummings J, Lee G, Mortsdorf T, Ritter A, Zhong K (2017). Alzheimer's disease drug development pipeline: 2017. Alzheimers Dement (N Y).

[8] Panza F, Lozupone M, Logroscino G, Imbimbo BP (2019). A critical appraisal of amyloid-β-targeting therapies for Alzheimer disease. Nat Rev Neurol.

[9] Martin EI, Ressler KJ, Binder E, Nemeroff CB (2009). The neurobiology of anxiety disorders: brain imaging, genetics, and psychoneuroendocrinology. Psychiatr Clin North Am.

[10] Ravindran LN, Stein MB (2010). The pharmacologic treatment of anxiety disorders: a review of progress. J Clin Psychiatry.

[11] Fisher RS, Acevedo C, Arzimanoglou A (2014). ILAE official report: a practical clinical definition of epilepsy. Epilepsia.

[12] Kotov AS (2020). [Remissions and relapses in epilepsy]. Zh Nevrol Psikhiatr Im S S Korsakova.

[13] Engel J Jr (2018). The current place of epilepsy surgery. Curr Opin Neurol.

[14] Moghaddam B, Javitt D (2012). From revolution to evolution: the glutamate hypothesis of schizophrenia and its implication for treatment. Neuropsychopharmacology.

[15] Fornito A, Zalesky A, Pantelis C, Bullmore ET (2012). Schizophrenia, neuroimaging and connectomics. Neuroimage.

[16] Acosta FJ, Hernández JL, Pereira J, Herrera J, Rodríguez CJ (2012). Medication adherence in schizophrenia. World J Psychiatry.

[17] Carpenter WT, Bustillo JR, Thaker GK, van Os J, Krueger RF, Green MJ (2009). The psychoses: cluster 3 of the proposed meta-structure for DSM-V and ICD-11. Psychol Med.

[18] American Psychiatric Association (2013). Diagnostic and Statistical Manual of Mental Disorders, Fifth Edition.

[19] Maenner MJ, Shaw KA, Baio J (2020). Prevalence of autism spectrum disorder among children aged 8 years—autism and developmental disabilities monitoring network, 11 Sites, United States, 2016. MMWR Surveill Summ.

[20] Zikopoulos B, Barbas H (2013). Altered neural connectivity in excitatory and inhibitory cortical circuits in autism. Front Hum Neurosci.

[21] Rubenstein JL, Merzenich MM (2003). Model of autism: increased ratio of excitation/inhibition in key neural systems. Genes Brain Behav.

[22] Chaste P, Leboyer M (2012). Autism risk factors: genes, environment, and gene-environment interactions. Dialogues Clin Neurosci.

[23] Hyman SL, Levy SE, Myers SM (2020). Identification, evaluation, and management of children with autism spectrum disorder. Pediatrics.

[24] Pierce K, Gazestani VH, Bacon E (2019). Evaluation of the diagnostic stability of the early autism spectrum disorder phenotype in the general population starting at 12 months. JAMA Pediatr.

[25] Wu Z, Fang Y (2014). Comorbidity of depressive and anxiety disorders: challenges in diagnosis and assessment. Shanghai Arch Psychiatry.

[26] Lichtenstein P, Yip BH, Björk C, Pawitan Y, Cannon TD, Sullivan PF, Hultman CM (2009). Common genetic determinants of schizophrenia and bipolar disorder in Swedish families: a population-based study. Lancet.

[27] Vossel KA, Beagle AJ, Rabinovici GD (2013). Seizures and epileptiform activity in the early stages of Alzheimer disease. JAMA Neurol.

[28] Jack CR Jr, Bennett DA, Blennow K (2018). NIA-AA research framework: toward a biological definition of Alzheimer’s disease. Alzheimers Dement.

[29] Hyman SE (2012). Revolution stalled. Sci Transl Med.

[30] Lakeman R (2014). Saving normal an insider’s revolt against out-of-control psychiatric diagnosis, DSM-5, big pharma, and the medicalization of ordinary life. Psychosis.

